# PrimerX: A Bayesian Multistage Cohort Embedded Randomised Trial to Evaluate the Role of Deferred Local Therapy of the Primary Tumour in Combination with Immune Checkpoint Inhibitor–based First-line Therapy in Metastatic Renal Cell Carcinoma Patients

**DOI:** 10.1016/j.euros.2024.09.002

**Published:** 2024-10-14

**Authors:** Orlane Figaroa, Patricia Zondervan, Rob Kessels, Johannes Berkhof, Maureen Aarts, Paul Hamberg, Maartje Los, Djura Piersma, Bart Rikhof, Britt Suelmann, Metin Tascilar, Astrid van der Veldt, Paul Verhagen, Hans Westgeest, Hilin Yildirim, Axel Bex, Adriaan Bins

**Affiliations:** a Department of Medical Oncology, Amsterdam University Medical Centres, University of Amsterdam, Amsterdam, The Netherlands; b Department of Urology, Amsterdam University Medical Centres, University of Amsterdam, Amsterdam, The Netherlands; c Cancer Center Amsterdam, Amsterdam University Medical Centres, Amsterdam, The Netherlands; d Julius Center for Health Sciences and Primary Care, Department of Data Science and Biostatistics, University Medical Centre Utrecht, Utrecht University, Utrecht, The Netherlands; e Department of Epidemiology and Data Science, Amsterdam UMC location Vrije Universiteit, Amsterdam, The Netherlands; f Department of Medical Oncology, Maastricht University Medical Centre, Maastricht, The Netherlands; g Department of Internal Medicine, Franciscus Gasthuis, Vlietland, Rotterdam/Schiedam, The Netherlands; h Department of Medical Oncology, St. Antonius Ziekenhuis, Nieuwegein/Utrecht, The Netherlands; i Department of Medical Oncology, Medical Spectrum Twente, Enschede, The Netherlands; j Department of Medical Oncology, Leeuwarden Medical Center, Leeuwarden, The Netherlands; k Department of Medical Oncology, Utrecht University Medical Centre, Utrecht, The Netherlands; l Department of Medical Oncology, Isala Medical Centre, Zwolle, The Netherlands; m Department of Medical Oncology, Erasmus Medical Center, Rotterdam, The Netherlands; n Department of Urology, Erasmus Medical Center, Rotterdam, The Netherlands; o Department of Medical Oncology, Amphia Medical Centre, Breda, The Netherlands; p Department of Research and Development, Netherlands Comprehensive Cancer Organisation, Utrecht, The Netherlands; q Department of Urology, Antoni van Leeuwenhoek Hospital-The Netherlands Cancer Institute, Amsterdam, The Netherlands; r Specialist Centre for Kidney Cancer, The Royal Free London NHS Foundation Trust, London, UK

**Keywords:** Checkpoint inhibitors, Deferred cytoreductive nephrectomy, Immunotherapy, Renal cell carcinoma

## Abstract

**Background:**

Historically, patients with metastatic renal cell carcinoma (mRCC) have been offered upfront cytoreductive nephrectomy (CN) followed by systemic therapy. Currently, CN is no longer the standard of care (SOC) based on the randomised phase 3 CARMENA study performed in the vascular endothelial growth factor receptor tyrosine kinase inhibitor era. With the advent of immune checkpoint inhibitor (ICI) combination therapy in first line, the role of CN needs to be reassessed. There is indirect evidence from small retrospective series that deferred CN after ICI combination therapy may lead to better outcomes. To reassess the role of CN, we designed PrimerX, a randomised controlled trial following the Trial within Cohorts (TwiCs) study design. The primary objective of this study is to re-evaluate the benefit of deferred local treatment in the current era of immunotherapy.

**Study design:**

This PrimerX study has been designed as a TwiCs study within the Dutch Prospective Renal Cell Carcinoma (PRO-RCC) cohort. The PRO-RCC cohort includes patients with mRCC and nonmetastatic RCC, and has been set up for prospective collection of long-term clinical data and as an infrastructure for initiating TwiCs studies. The PrimerX TwiCs trial follows a Bayesian adaptive multistage design to allow for early discontinuation due to futility or efficacy. PrimerX has appropriate ethics approval and is registered at clinical.trials.gov (NCT05941169).

**End points:**

The primary clinical endpoint is overall survival, defined as the time from randomisation to death from any cause. The secondary endpoint is the objective response rate within the primary tumour prior to local therapy, as assessed by a computed tomography scan.

**Patients and methods:**

A maximum of 700 patients with synchronous mRCC and absence of progression at metastatic sites following at least 6 mo of standard first-line ICI combination therapy will be assigned randomly to receive local treatment of the primary tumour (experimental arm) or SOC (control arm). The experimental intervention consists of (partial) CN, any form of ablative local therapy, or magnetic resonance imaging guided ablative stereotactic radiotherapy, performed within 6 mo and 1.5 yr after the start of systemic treatment.

## Take Home Message

To reassess the role of deferred cytoreductive nephrectomy in the current era of immunotherapy, we designed PrimerX, a randomised controlled trial following the Trial within Cohorts (TwiCs) study design.

## 1 Introduction

Renal cell carcinoma (RCC) accounts for 3% of adult malignancies and 95% of renal tumours. In 2020, >430 000 new cases of RCC were diagnosed globally, with almost 180 000 new deaths [1], [2]. According to recent epidemiological data, between 10% and 20% of patients diagnosed annually with RCC present with synchronous metastatic disease and the primary tumour in place [3].

Cytoreductive nephrectomy (CN) was introduced at a time when first-generation immunotherapy was the only systemic treatment available and the median overall survival (OS) for metastatic RCC (mRCC) was <1 yr [4]. In this context, the benefit of CN was estimated to be around 6 mo, based on two trials that formally evaluated the benefit of CN. In both studies, patients were randomised to receive interferon-α (IFN-α) + CN or IFN-α alone. Both studies reported a benefit: a 3-mo survival benefit in a larger US study and a 10-mo survival benefit in a smaller European study [5], [6]. Several hypotheses were proposed to explain why CN might have a beneficial effect: removal of the “immunological sink” [7], reduced production of growth factors and cytokines by the tumour in situ [8], delayed metastatic progression [9], and nephrectomy-activated azotaemia [10] were among the proposed potential mechanisms.

Since then, two trials have been conducted to assess the role and sequence of CN in combination with vascular endothelial growth factor receptor (VEGFR)-targeted therapy.

Firstly, the CARMENA trial randomised patients to receive CN plus sunitinib versus sunitinib alone to assess the benefit of CN in patients receiving vascular endothelial growth factor–targeted therapy [11]. Secondly, the SURTIME trial randomised patients to receive CN followed by sunitinib versus sunitinib followed by CN (“deferred CN”), investigating the role of preoperative VEGFR-targeted therapy in combination with cytoreduction [12]. Together, the CARMENA and SURTIME analyses provide strong evidence that immediate CN has no benefit and should not be performed in intermediate- and poor-risk Memorial Sloan Kettering Cancer Center patients who require sunitinib or an equivalent VEGFR tyrosine kinase inhibitor (TKI), with, respectively, 43% and 11% survival in poor-risk patients. The OS of patients who received immediate CN in both studies was shorter than that of patients who received immediate sunitinib, although not statistically significant.

Notably, the favourable survival outcome observed in the CARMENA control-arm subgroup, which received deferred CN despite randomisation in the control arm, suggests that there may not be an immediate need to perform early CN [11]. In this subgroup, CN was performed at a median duration of 11 mo of treatment (ranging from 7 to 87 mo), suggesting that confirmation of an on-going response through subsequent imaging could serve as a practical approach for patient selection. Despite the CARMENA and SURTIME studies, the question remains whether mRCC patients receiving immediate sunitinib benefit from deferred CN.

The introduction of first-line immunotherapy with nivolumab and ipilimumab in 2019, based on the CheckMate-214 trial, has led to a paradigm shift in the treatment of clear cell mRCC [13]. The study demonstrated superior OS with nivolumab and ipilimumab to that with sunitinib in clear cell mRCC patients with International Metastatic RCC Database Consortium (IMDC) intermediate or poor prognosis. In these patients, most objective responses occurred by 16 wk. In a 5-yr update, the objective response rate in intermediate and poor IMDC risks was 42%, with 11% developing a complete radiological response [14]. In these risk groups, immune checkpoint inhibitor (ICI) combination therapy is now the standard of care (SOC), with sunitinib and other VEGFR TKI monotherapies being reserved for those who cannot tolerate or do not have access to ICIs. Based on the inclusion of up to 30% of patients without prior nephrectomy in the pivotal ICI trials, the major guidelines continue to recommend immediate systemic therapy for patients with synchronous mRCC [15], [16].

With increased availability of ICI combination therapy, a growing number of these patients develop prolonged systemic disease control and are being offered deferred CN [17]. However, the evidence for the benefit of CN in the context of ICI combination treatment is limited. Retrospective series describe improved survival following ICI combination therapy and deferred CN [18]. Complete pathological responses of the primary tumour have been described in 11–13% of patients treated previously with ICIs [19], [20]. Withdrawal of systemic therapy with treatment-free periods of several years has been reported in patients who had a complete response of metastatic sites to first-line ICIs and were rendered disease free by deferred CN [21], [22]. Our own experience with 21 deferred CN cases after nivolumab plus ipilimumab showed very few desmoplastic tissue changes and adverse events, and three (14%) patients with a complete pathological response [19]. Additional indirect evidence comes from genotypic studies investigating metastatic phenotypes in RCC, which have demonstrated that more aggressive clones may develop in the primary tumour over time, potentially leading to rapid progression and early mortality [13]. These potential benefits require prospective randomised testing against the current standard of systemic therapy alone.

Contemporary retrospective data reveal that the majority of patients (up to 75%) with synchronous metastatic disease no longer undergo CN as part of their management [23], [24]. However, the population without nephrectomy has been a minority in the pivotal ICI combination therapy trials (16.6–30.1%) [16], and there is considerable uncertainty regarding how to manage patients in this setting. To reassess the role of deferred CN in the current era of immunotherapy, we designed PrimerX, a randomised controlled trial (RCT) following the Trial within Cohorts (TwiCs) study design.

## 2 Methods

### 2.1 Study design

**Fig. 1:**
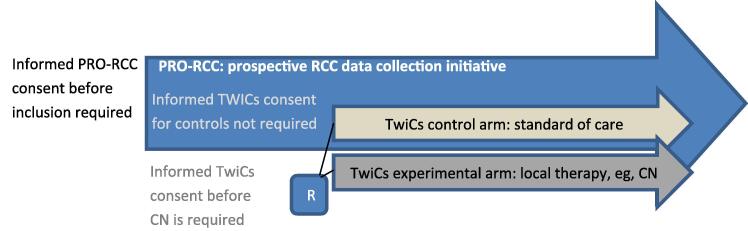
Embedding of PrimerX TwiCs study within PRO-RCC. CN = cytoreductive nephrectomy; PRO-RCC = Prospective Renal Cell Carcinoma; RCC = renal cell carcinoma; TwiCs = Trial within Cohorts.

The PrimerX study is conducted within the Dutch Prospective Renal Cell Carcinoma (PRO-RCC) cohort [25]. The PRO-RCC cohort includes patients with metastatic and nonmetastatic renal cancer, and has been designed for prospective collection of long-term clinical data, patient-reported outcome measures, and patient-reported experience measures. Furthermore, the PRO-RCC cohort can be used as an infrastructure for initiating TwiCs studies [26]. The PrimerX study follows this TwiCs study design (Fig. 1). In addition, a Bayesian adaptive multistage design is applied to allow for early stopping for futility or efficacy. Synchronous mRCC patients will be randomised to local therapy (deferred CN or any form of local ablative therapy; experimental intervention) or to a control arm that receives the SOC, in principle without CN. The primary objective is to reassess the benefit of deferred CN in the current immunotherapy era, by a preset analysis of the OS clinical endpoint.

### 2.2 Primary and secondary endpoints

The primary clinical endpoint is OS, defined as the time from randomisation to death of any cause. The main secondary endpoint is the objective response rate in the primary tumour before local therapy, assessed by a computed tomography scan. Other secondary endpoints are long-term follow-up of quality of life (QoL) using the Dutch validated European Organisation for Research and Treatment of Cancer (EORTC) Quality of Life Questionnaire (QLQ-C30) [27] and Clavien-Dindo classification of surgical morbidity [28]. Both follow-up of QoL and surgical morbidity are part of the standard PRO-RCC data collection schedule.

### 2.3 Exploratory outcomes

For one of the exploratory outcomes, fresh-frozen and paraffin-embedded tissue of pretreatment biopsies and post-treatment tissue obtained at either partial or total nephrectomy will be collected, as well as peripheral blood before and during treatment, to analyse the immune compartments in the peripheral blood and in the tumour, especially tumour infiltrating lymphocytes and myeloid-derived suppressor cells (MDSCs). In detail, we will examine the intratumour alteration of tumour infiltrating CD8, CD4, and NK(T) cells (CD56+ and CD3+/–) and the distribution of these NK cells in the tumour. Furthermore, Treg (CD3+CD4+FoxP3+), tumour-associated macrophages (TAM, CD68+, CD16+/–, and CD163+/–), and MDSCs (CD11b+, HLA-DR, and CD33+), differentiating between pro- and anti-inflammatory M1 and M2 subtypes, will be assessed. In addition, genomic profiling (Illumina assay) and the analysis of effects on angiogenesis will be investigated.

### 2.4 Eligibility criteria

At enrolment in the PRO-RCC cohort, patients are asked to provide informed consent for prospective collection of clinical, survival, and QoL data. In addition, on entry in the cohort, we ask patients to consent for possible future randomisation in TwiCs studies (staged informed consent procedure) [29]. Herein, patients are informed that, when a TwiCs study to investigate the efficacy of a new experimental intervention is initiated, they will be offered that experimental intervention if they are selected randomly for the experimental intervention group. They are also advised that they will not be informed about the TwiCs study when selected randomly for the control group but that their data will be used as in the trial context. Patients within the PRO-RCC cohort who meet the PrimerX inclusion criteria, as mentioned in Table 1, form a subcohort of eligible patients. Within this subcohort, patients without progression at metastatic sites, with the primary tumour in site (irrespectively of the size of the tumour), following at least 6 mo of any of the standard first-line ICI combination therapies, are allocated randomly to either the experimental arm or the control arm in a 1:1 ratio, until the calculated sample size is reached or early stopping rules are met. Patients who have progression in the primary tumour only are also eligible for trial participation. Since patients are randomised at different time points, between 6 and 18 mo after the start of systemic treatment, the timing of randomisation is added as a variable when conducting the analysis.

**Table 1:** Inclusion criteria

Male or female patients age ≥18 yr
Signed and written informed consent obtained; note: written and signed informed consent will be obtained before any study procedures, including study‐specific-screening procedures
Informed consent obtained for being offered future experimental interventions within the PRO-RCC project
Histologically confirmed diagnosis of metastatic clear cell, papillary, or chromophobe renal cell carcinoma of intermediate to poor risk, including sarcomatoid features
World Health Organization performance status of 0–1
Surgical candidates based on surgeon and anaesthetist assessment
Treatment with an IO combination (IO + IO or IO + TKI) as standard of care for metastatic RCC for at least 6 mo
Absence of progression at metastatic sites at the time of identification (6 mo after the start of systemic first-line treatment)
Primary tumour in situ
Participation in the PRO-RCC prospective cohort

IO = immune oncology; PRO-RCC = Dutch Prospective Renal Cell Carcinoma; RCC = renal cell carcinoma; TKI = tyrosine kinase inhibitor.

Patients who are randomised to the experimental arm will be counselled by their treating physician for participation in the experimental arm. If these patients accept the intervention, they are asked to sign an additional informed consent form containing all information regarding the PrimerX study, as part of the staged informed consent procedure [29]. Patients who refuse the offered experimental intervention will continue to receive the SOC. Patients randomised to the control arm will receive no additional consent for the TwiCs study, and thus not informed about the experimental intervention or about their participation in the study as controls (Fig. 1), based on their consent upon entry in the cohort. Patients randomised to the control arm will receive the SOC.

### 2.5 Experimental intervention

**Fig. 2:**
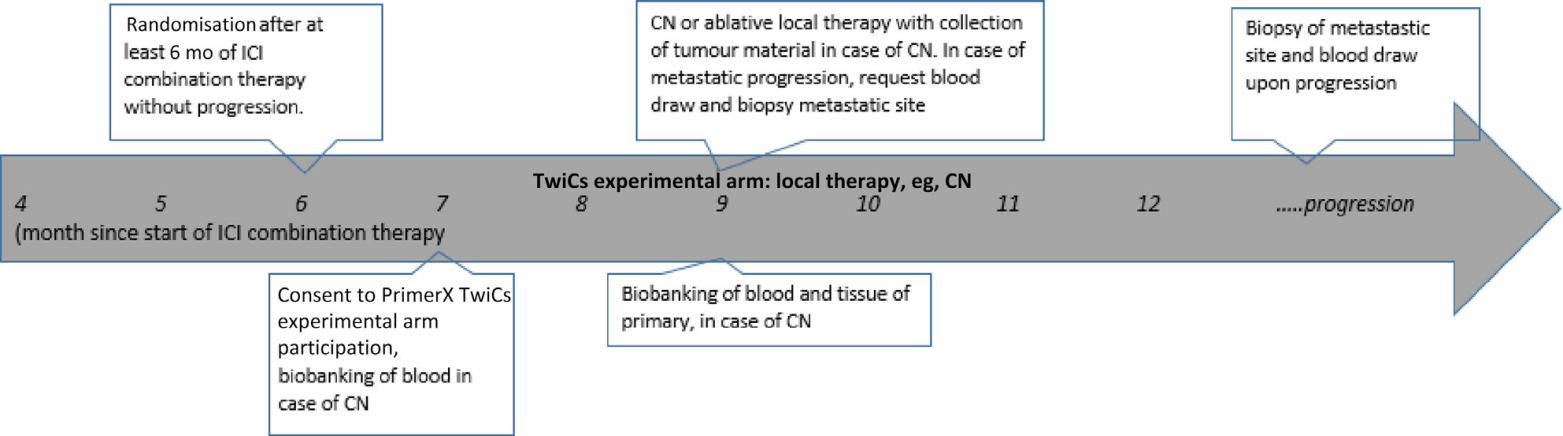
Overview of PrimerX TwiCs experimental arm including randomisation, follow-up, and procedure in case of progression. CN = cytoreductive nephrectomy; ICI = immune checkpoint inhibitor; TwiCs = Trial within Cohorts.

The experimental intervention consists of (partial) CN or any ablative local therapy between 6 mo and 1.5 yr after the start of systemic treatment. All types of ablative local therapies are allowed as a substitute for CN, including magnetic resonance imaging–guided stereotactic radiation. The patients’ physician chooses the treatment plan and the modality used for tumour ablation. Randomisation occurs after at least 6 mo of ICI combination therapy, as stated in Figure 2. Patients who discontinue their first-line treatment due to progression at metastatic sites in the timeframe between randomisation and local therapy are disqualified for local treatment but continued in the TwiCs study in order to be part of the intention-to-treat (ITT) analysis. The same applies for patients who are randomised to the experimental arm but who refuse local treatment. All these patients will be treated according to the SOC. Patients in the experimental arm will be asked for additional sampling of blood after the local intervention.

### 2.6 SOC treatment

Patients randomised to the control arm will receive first-line ICI combination therapy according to current guidelines, which do not routinely recommend CN in this setting [20]. Patients with controlled systemic disease but progression of the primary tumour can be offered deferred CN at the discretion of the treating physician. Likewise, patients with a complete response at metastatic sites who can be rendered disease free by deferred CN are not excluded from an intervention if the treating physician considers nephrectomy in the patient’s best interest. These patients remain in the TwiCs study for the ITT analysis.

### 2.7 Sample size calculation and Bayesian adaptive design

Estimation of the median OS of the selected subcohort for PrimerX is not trivial. Based on the CARMENA trial, synchronous mRCC patients treated with sunitinib without CN have median OS of 18.4 mo. All CARMENA patients were treated with VEGFR TKIs only, as ICI combination therapy was not available at the time. In these patients, a reduction in the hazard of death of 20–40% (if they would have been treated with ICI combination therapy, based on the CheckMate-214 study) would have resulted in median OS of 24–28 mo. This is in line with the post hoc OS analysis of mRCC patients with the primary tumour in place in the CM-214 study [30]. This demonstrated median OS of 26.1 mo (95% confidence interval [CI] 14–35 mo) for the ipilimumab + nivolumab arm versus 14.3 mo (95% CI 9.7–23 mo) for the sunitinib arm.

We therefore assume that the median OS for IMDC intermediate/poor-risk synchronous mRCC patients may be up to 28 mo. As in the PrimerX study patients with early systemic disease progression (within 6 mo) are excluded from participation, we estimate that the remaining patients have median OS of 36 mo, when treated with the SOC. The median OS of 36 mo comes down to an event rate per week (lambda) of 1 – (0.5^[1/156 wk]) = 0.0044. We believe that a meaningful deferred local therapy should reduce the hazard of death by at least 25% compared with the SOC (hazard ratio [HR] of 0.75), that is, a 12-mo survival benefit. This would balance the additional treatment burden of local therapy and would result in a median excess in OS of 48 mo in the experimental arm. Furthermore, we estimate that one out of six patients (15%) who are randomised to the experimental arm will refuse any form of local therapy and will therefore be treated by the SOC instead. This noncompliance reduces the measured benefit in an ITT analysis. In addition, a number of patients in the control arm will undergo CN. This also reduces the measured benefit. Based on our 2018–2020 IKNL dataset, we estimate that such “control dilution” will occur in approximately 7% of the cases, as over this period 60 out of 754 synchronous mRCC patients underwent deferred CN. Both noncompliance and control dilution reduce the anticipated HR from 0.75 to 0.7975. After correcting for control dilution, the lambda of the control arm drops from 0.0044 to 0.0043.

The maximum required number of patients has been set at 700 (350 per arm) based on conventional power calculation. However, the trial uses a Bayesian adaptive multistage trial design, where at five subsequent interim analyses, evaluation of futility and success takes place using a Bayesian model with conservative stopping boundaries. Therefore, the actual power has been determined by means of simulation, performed using FACTS software (FACTS 7; Berry Consultants LLC, Austin, TX, USA). The Bayesian interim analyses take place after 250, 350, 450, 550, and 650 patients have been randomised. Thereby, there is an opportunity to arrive at an early “success or fail” prediction based on the posterior distribution, after randomising only a part of the maximum sample size. Parameters (ie, stopping boundaries; see Table 2) of the Bayesian interim analyses were chosen such that the frequentist operating characteristics of the trial yield the desired overall type I error rate (falsely rejecting the null hypothesis) of 0.05 and type II error rate (failing to reject the null hypothesis when false) of 0.2 (power of 0.80). These characteristics were evaluated using in silico trial simulations. Importantly, the first two interim checks are not Bayesian and not part of the simulation study. Interim checks 1 and 2 are included to monitor the actual noncompliance rate in the experimental arm and the control-dilution rate in the control arm.

**Table 2:** Stopping boundaries

Interim	*N*	Probability limit of HR <0.9, for futility	Probability limit of HR <0.9, for success
1	75	If noncompliance is >15% or control-dilution >7%, the cause will be investigated and addressed in recruiting centres	
2	750	If the HR corrected for control dilution and noncompliance is >0.85, the trial will stop; if between 0.79 and 0.85, then the DSMB may decide to adjust the maximum sample size	
3	250	<0.03	>0.99
4	350	<0.06	>0.98
5	450	<0.12	>0.96
6	550	<0.24	>0.92
7	650	<0.36	>0.84

DSMB = Data and Safety Monitoring Board; HR = hazard ratio.

In the Bayesian model, we used weakly informative conjugate gamma priors for the control hazard rate and weakly informative conjugate normal priors for the log-HR. Parameters for the prior distribution of the hazard rate were chosen such that these mirror the hazard rate of the control arm. The prior distribution for the log-HR had a mean of 0 and standard deviation of 5, which is considered to be a weak prior. All scheduled interim analyses estimate the Bayesian posterior probability of an HR of <0.9, and the corresponding stopping boundaries are listed in Table 2. For example, after 350 patients have been randomised, the trial will stop for futility if the posterior probability for HR <0.9 drops below 0.06. With these settings, 10 000 trial simulations were performed in FACTS (5000 for the scenario under the null hypothesis with HR = 1 and 5000 under the alternative scenario with HR = 0.7975). The results of these simulations revealed that the Bayesian empirical type I error rate of the trial was 5.6% and that the empirical power was 76%, to detect a Bayesian posterior probability of ≥75% for an HR of <0.9, during the final analysis under the alternative hypothesis. Importantly, the final one-sided frequentist log-rank test had a power of 79.8% under the alternative hypothesis that the HR is <1 (as opposed to <0.9 in the Bayesian interims) and a frequentist type I error rate of 4.9%. The average sample size was 548.6 in case of an HR of 1 and 596.8 in case of an HR of 0.7975. Notably, a standard two-arm trial without interim analyses would require 700 patients in total.

We also ran 10 000 simulations using an HR of 0.84, simulating a weaker HR due to a higher control dilution and noncompliance (ie, respectively, 15% and 25%) and thereby a lambda of 0.0042 in the control arm. In this scenario, we modelled a maximum of 1000 patients, with interim analyses of 350, 450, 550, 650, 750, 850, and 950 patients and adjusted boundaries as summarised in Table 2. All other settings were kept unchanged. The results of this scenario are still satisfactory, and can be implemented after the measurement of control dilution and noncompliance at the second interim. The Data and Safety Monitoring Board (DSMB) will convey after this interim and is responsible for the decision whether to adapt the maximum sample size of the study at this point. Under the HR = 0.84 assumption, the overall Bayesian type I error rate was 4.3% and the empirical power was 69.1% to detect a posterior probability of 70% for HR >0.9. The average sample size was 869 patients under the null scenario (HR = 1) and 892.4 under the alternative scenario (HR = 0.84). The final frequentist log-rank test had a power of 78.6% and a type I error rate of 5.3%.

### 2.8 Analysis plan

Analyses are conducted on an ITT basis. An ITT analysis in a TwiCs study represents the average causal effect of offered treatment, and the intercurrent event noncompliance in the experimental intervention arm is handled according to the treatment policy strategy [31]. At the interim analyses, provisional efficacy will be concluded when the posterior probability of HR <0.9 exceeds the efficacy stopping boundaries, as listed in Table 2. If this happens at an interim analysis, recruitment is stopped, but follow-up is continued. The trial will stop for futility as soon as an interim analysis reveals that the posterior probability for an HR of <0.9 drops below the futility stopping boundaries, as listed in Table 2.

If the maximum sample size has been reached or accrual has been halted based on the efficacy stopping rules, a final analysis will be conducted using the log-rank test for OS after the completion of 5-yr follow-up for all patients. Efficacy of the experimental intervention at the final analysis will be concluded when the one-sided *p* value of this final log-rank falls below 0.05. The OS curve will be constructed using the Kaplan-Meier method. In addition, a per-protocol analysis will be performed. Moreover, ITT and per-protocol subgroup analyses will be performed for subgroups based on the type of local treatment and type of systemic treatment.

In addition, Fisher exact test will be used to compare the treatment arms on objective response rate and surgical morbidity. Follow-up QoL will be assessed by linear mixed models adjusting for baseline score, including treatment group and time as fixed covariates and treating patients at random.

## 3 Discussion

The SURTIME and CARMENA trials were performed prior to the availability of ICIs, when VEGFR TKIs were the mainstay of systemic treatment. Given the benefit of CN in the setting of first-generation immunotherapy, it is conceivable that both trials underestimated the benefit of CN in the absence of immunotherapy. Hence, the benefit of deferred CN or local therapy needs to be reassessed in the context of ICI combination therapy.

The current guidelines from the European Association of Urology suggest that in patients showing a clinical response to ICI-based combinations, subsequent CN may be considered [20]. However, it is important to note that this recommendation is based on weak evidence. Therefore, the purpose of this study is to either confirm or refute the existing limited evidence.

Performing CN after prior ICI combination therapy appears to be safe with pathologically favourable tumour characteristics, although the numbers reported are limited. Considering the limited risks involved, the significance of addressing this research question outweighs the potential drawbacks.

This study is designed as a TwiCs study, which has some appealing characteristics compared with the classical RCT. Classical RCTs are often confronted with slow recruitment, a disappointment bias in patients randomised to the control arm leading to potential dropout, and limited external validity. The TwiCs trial design was introduced to overcome these shortcomings [26]. Since patients in the control arm are not informed about the experimental deferred CN, they are unlikely to be discouraged by not being offered a new treatment, thus preventing dropout. In addition, the observational cohort represents the population more adequately because cohort studies are generally less selective when including patients. This can prevent the selection bias when recruiting patients for a TwiCs study (see, eg, the study of Gal et al [32]).

Furthermore, this TwiCs trial incorporates a Bayesian adaptive multistage trial design with a flexible number of patients to be included, ensuring that only patients who are actually needed to reach conclusions will enter the trial. In addition, the Bayesian approach for interim analyses is more flexible than the frequentist approach since early stopping does not affect Bayesian inference, and hence, multiplicity issues do not play a role [33]. To our knowledge, this is the first TwiCs study design that follows a Bayesian adaptive multistage trial design, thereby possessing multiple advantages and flexibilities compared with the classical way of conducting RCTs.

A challenge of a TwiCs study is the anticipated refusal rate in the experimental arm and how this affects the ITT effect and initial sample size calculations. Some previous TwiCs studies in oncology have been proved to underestimate the refusal rate, and sample sizes had to be increased during the trial [31]. This is also a potential risk for the current trial, which is why we have incorporated two early interim checks to monitor the refusal rates and prematurely stop the trial in case the refusal rate is much larger than expected. In addition, we have simulated a trial scenario in a situation where initial refusal rates are underestimated, in which we follow the recommendation to evaluate trial scenarios under different refusal rate assumptions [34].

We took great care to establish median OS estimates for the sample size calculation. The majority of patients presenting with mRCC and the primary tumour in place belong to the IMDC intermediate- and poor-risk categories. In a recent 5-yr update of IMDC intermediate- and poor-risk patients treated with nivolumab and ipilimumab, the median OS was 47 mo. However, for patients with synchronous mRCC, this is likely an overestimation, as 78% of the patients in the CheckMate-214 trial had metachronous disease. The HR for OS in that trial was 0.68 in favour of the combination, resulting in a 32% reduction in the hazard of death compared with sunitinib. Based on the median OS results in the sunitinib-alone arm in the CARMENA trial, the median OS can be deduced for a population of patients with synchronous mRCC treated with nivolumab and ipilimumab. Two other trials investigating deferred CN in combination with ICI therapy in the USA and Scandinavia, using a classical RCT design, used similar median OS estimates of 24 mo [35], [36]. However, they underestimated the survival gain achieved by excluding patients with no clinical benefit following ICI therapy from randomisation, which reduces the event rate and requires larger sample sizes.

PrimerX is not designed to investigate the role of deferred CN in combination with a specific first-line standard, nor is it suited to study the optimal time point for an intervention. However, the lessons learned from the EORTC SURTIME trial demonstrate that complexity in surgical trials jeopardises accrual. Evidence from CARMENA and SURTIME suggests that deferred CN can be considered upon documentation of a sustained response to systemic therapy rather than at a specific point in time. In addition, we believe that removal of the primary tumour should be investigated as a concept in the multimodality management of mRCC rather than as a specific approach. While nephrectomy has evolved as the gold standard for cytoreduction, recent data suggest that thermal ablation or stereotactic ablative body radiotherapy may be equally effective in smaller tumours. As ICI combination therapy may downsize primary tumour diameters by >70%, PrimerX specifically allows alternative local therapies in the experimental arm.

## 4 Ethics approval and consent to participate

This RCT study received approval by the medical ethical review board of the Amsterdam University Medical Center, The Netherlands, in 2024 (2023.0428). All methods will be conducted in accordance with the ethical standards of the declaration of Helsinki and will be in accordance with relevant guidelines and regulations. Informed consent will be obtained from all patients before inclusion.

  ***Author contributions*:** Orlane Figaroa had full access to all the data in the study and takes responsibility for the integrity of the data and the accuracy of the data analysis.

  *Study concept and design*: Figaroa, Bins, Bex.

*Acquisition of data*: Figaroa, Bins, Bex.

*Analysis and interpretation of data*: Figaroa, Bins, Bex.

*Drafting of the manuscript*: Figaroa, Zondervan, Kessels, Berkhof, Aarts, Hamberg, Los, Piersma, Rikhof, Suelmann, Tascilar, van der Veldt, Verhagen, Westgeest, Yildirim, Bex, Bins.

*Critical revision of the manuscript for important intellectual content*: Figaroa, Zondervan, Kessels, Berkhof, Aarts, Hamberg, Los, Piersma, Rikhof, Suelmann, Tascilar, van der Veldt, Verhagen, Westgeest, Yildirim, Bex, Bins.

*Statistical analysis*: Figaroa, Bins, Bex, Berkhof, Kessels.

*Obtaining funding*: Figaroa, Bins, Bex.

*Administrative, technical, or material support*: Figaroa, Bins, Bex.

*Supervision*: Baard, Bins, Bex.

*Other*: None.

  ***Financial disclosures:*** Orlane Figaroa certifies that all conflicts of interest, including specific financial interests and relationships and affiliations relevant to the subject matter or materials discussed in the manuscript (eg, employment/affiliation, grants or funding, consultancies, honoraria, stock ownership or options, expert testimony, royalties, or patents filed, received, or pending), are the following: None.

  ***Funding/Support and role of the sponsor*:** This work was supported by KWF, Cure for Cancer.
